# Frailty as a Predictor of Post-Traumatic Stress Disorder After Advance Care Planning Communication Intervention by Trained Care Managers in Long-Term Care Service Users in Japan: A Secondary Analysis

**DOI:** 10.3390/jpm15040159

**Published:** 2025-04-21

**Authors:** Mariko Miyamichi, Kyoko Oshiro, Shozo Okochi, Noriyasu Takeuchi, Tomoe Nakamura, Terumi Matsushima, Masako Okada, Yoshimi Kudo, Takehiro Ishiyama, Tomoyasu Kinoshita, Hideki Kojima, Mitsunori Nishikawa

**Affiliations:** 1 Yorozu Soudanjyo Co., Ltd., Akadouji–cho, Oohori, 18, Konan-City 483-8221, Aichi, Japan; 2 Wabisabi Home Care Support Office, Tsutsujigaoka, 3-11-25, Chita-City 478-0054, Aichi, Japan; 3 Social Welfare Corporation Yotsubakai, Tokubo, 504, Kurashiki-City 710-0011, Okayama, Japan; 4 Magokoro Home Care Support Office, Shinchikajihazama, 70, Chita-City 478-0017, Aichi, Japan; 5 Care Plan Tsuyukusa Home Care Support Office, Wakata 3-112, Nagoya-City 458-0034, Aichi, Japan; 6 Kagayaki Home Care Support Office, Tanto-cho, Dosakiazagounishi, 740-1, Ichinomiya-City 491-0825, Aichi, Japan; 7 Suito Home Care Support Office, Nisigata, 1538-1, Kuwana-City 511-0864, Mie, Japan; 8 Nichii Care Center Koudunomori Home Care Support Office, Koudunomori, 5-21-2, Narita-City 286-0048, Chiba, Japan; 9 Social Medical Corporation Dohoku Kinsho Soya Clinic Designated Home Care Support Office, 3-6-5 Suehiro, Wakkanai-City 097-0001, Hokkaido, Japan; 10 National Center for Geriatrics and Gerontology, Morioka-cho, 7-430, Obu-City 474-8511, Aichi, Japan; 11 Aioi Geriatric Health Services Facility, Ogawa Higashikomeda, 16, Higashiura-cho, Chita-gun 470-2102, Aichi, Japan

**Keywords:** advance care planning, case managers, frailty, personalized care, post-traumatic stress disorder

## Abstract

**Background/Objectives**: Advance care planning is essential in a community; however, intervention studies by care managers remain scarce. This study aims to determine the relationship between frailty and post-traumatic stress disorder among long-term care service users (hereinafter referred to as “users”) following advance care planning conversations with their care managers. **Methods**: We conducted a secondary analysis using raw data from the Japanese University Hospital Medical Information Network Study No. 000048573, published on 23 September 2024. In this previous study, trained care managers provided advance care planning conversation interventions to 30 users. Care managers conducted a convenience sample of 30 mentally and physically stable users who were 65 years old or older, had a family member or healthcare provider assigned, and had never used ACP. Our analysis in the present study focuses on the Clinical Frailty Scale and Impact of Events Scale-Revised, both of which measure post-traumatic stress disorder. **Results**: The Impact of Events Scale-Revised score was significantly higher in users with a clinical frailty score ≥ 5 compared to those with a clinical frailty score < 5. Logistic regression analysis, using the Impact of Events Scale-Revised as the objective variable, also revealed an association between a clinical frailty score ≥ 5 and a higher Impact of Events Scale-Revised. The four groups, selected through hierarchical cluster analysis for sensitivity analysis, demonstrated results consistent with the above analysis. **Conclusions**: The degree of post-traumatic stress disorder among users is associated with their degree of frailty following an advance care planning conversation with their care manager. Frailty in users may be a valuable predictor of stress related to advance care planning conversations. Users with a clinical frailty scale score ≥ 5 can be provided with more personalized care through more careful communication. University Hospital Medical Information Network Trial ID: 000048573.

## 1. Introduction

Advance care planning (ACP) is necessary for older adults in a community [1,2]. However, although the concept of ACP is ideal, its implementation is complex, resulting in a significant gap between the ideal and actual practice [3]. Furthermore, ACP is strongly influenced by cultural differences, and replicating ACP practices from Europe and the United States is challenging. This has led to difficulties in ACP progress [4].

In Japan, the Ministry of Health, Labor, and Welfare guidelines were significantly revised in 2018 to bridge the gap between the ideal and actual ACP. The guidelines previously positioned doctors, nurses, and social workers as the primary professionals promoting ACP; however, the revised version now includes care managers. Previously, the primary role of a care manager was to coordinate home care services under long-term care insurance. With this revision, ACP has been incorporated into the care manager’s responsibilities [5].

Following the revision of these guidelines, we developed an ACP training program—the ACPiece program—that is both appropriate and culturally relevant for care managers in Japan [6,7]. This program was developed owing to a lack of research on the role of care managers. Although reports on care managers in ACP are limited globally, previous studies have the potential for sharing ACP tasks with care managers [8]. These studies suggest that care managers can practice and improve their engagement with ACP, albeit imperfectly [9,10]. A Japanese study suggested the possibility of involving care managers in ACP within the context of multidisciplinary collaboration [11].

Subsequently, we published two papers on ACPiece. First, we reported an improvement in care managers’ positive attitudes toward dying patients before and after participating in the ACPiece program [12]. Second, we reported that ACP engagement improved, and that post-traumatic stress disorder (PTSD) was not a significant factor before and after the engagement of care managers in ACP conversations with long-term care service users [13]. Notably, the representative values (mean and median) of PTSD scores for all cases were below the cutoff values, although some cases exceeded the cutoff values [13]. This study was called the CMACP-PPP study [13,14].

The protocol [14] for this study [13] excluded cases where PTSD was expected following the ACP intervention. The ACPiece used in this study was designed to minimize the invasiveness of ACP communication using communication skills such as repetition and silence [6,7]. However, some long-term care service users still exhibited high PTSD scores [13]. We explored the factors contributing to high PTSD scores. Subsequently, we conducted a narrative review of the PubMed database using ACP- and PTSD-related search terms. We focused on frailty-related terms as emerging keywords during the search process. PTSD is a post-traumatic stress state, while frailty is a state in which the body and mind function poorly and become less resilient to stress with age [15].

After searching PubMed for frailty- and PTSD-related terms, we focused on two articles. These studies reported an interrelationship between frailty and PTSD [15], with frail patients being more likely to experience PTSD when evaluated 1 year after recovering from hospitalization due to COVID-19 [16]. Nevertheless, the number of studies is limited, highlighting the research gap in this field.

Furthermore, when ACP- and PTSD-related terms were used as search terms in the PubMed database, we focused on five articles. All five articles discussed the stress of the surrogate decision-maker rather than the long-term care service user. Specifically, some studies reported that ACP implementation improved PTSD in the surrogate [17,18,19], whereas others reported no relationship between ACP implementation and PTSD [20]. Additionally, some reports suggest that the tendency for PTSD among surrogate decision-makers in ACP is linked to personality traits [21]. This highlights a research gap regarding the PTSD of the user, rather than the surrogate decision-maker, following ACP implementation.

When searching PubMed using ACP and frailty as search terms, 11 articles were identified. Some studies did not thoroughly discuss the relationship between frailty and ACP, but highlighted that the Clinical Frailty Scale (CFS) predicts life expectancy [22,23], is a good indicator of ACP initiation [24,25], that higher CFS scores are associated with higher rates of ACP initiation [26], and that conservative treatment of ACP is more often chosen in the CFS-measured group [27].

Previous studies have reported that a mild degree of frailty is more likely to be associated with ACP implementation [28]; however, frailty evaluation alone does not necessarily lead to ACP implementation [29]. Among the studies that evaluated individuals’ wishes, one study [30] stated that the high-CFS group was more likely to focus on how to live rather than on how to die during ACP conversations compared with the low-CFS group. In contrast, another study [31] found no association between the degree of CFS and congruence between the wishes of the individual and those of the family. Another study [32] stated that frail older people must consider their present and future wishes on a continuum, as they seek to live well in the present. Although studies relating ACP to frailty are relatively abundant, their findings vary. We were motivated by new insights into the extent of frailty and PTSD: (1) after ACP communication, (2) by care managers, and (3) by the users themselves, rather than their surrogates.

Therefore, this study aims to clarify the relationship between frailty and PTSD in long-term care service users when care managers, who are responsible for coordinating home care services in the community, engage in ACP conversations with long-term care service users, in collaboration with multiple professionals.

## 2. Materials and Methods

### 2.1. Definition of ACP

As stated by the Japan Geriatrics Society, ACP is a process that supports people in making decisions and accords respect to each individual as a human being, regarding their future medical and long-term care needs [33].

### 2.2. Ethical Consideration

The study received approval from the Institutional Review Board of the National Center for Geriatrics and Gerontology (approval number: 1619). It adhered to the guidelines of the Declaration of Helsinki, as revised in Brazil in 2013. Care managers verbally explained the study to the participants, providing a written description, and obtained their written consent.

### 2.3. Study Design

This study conducted a secondary analysis of a comparative pre- and post-intervention study conducted by trained care managers. An approximately 1 h ACP communication intervention was conducted, adhering to the content of the developed ACPiece program. The ACPiece program is described in detail in previous reports [6,7,12,13]. This initiative includes a short lecture, hands-on training, scenario analysis, role-playing, and collaborative activities. It was created to assist Japanese long-term care service users in expressing their hidden emotions and viewing care managers as empathetic partners. This is especially important in Japan, where genuine emotions are frequently unexpressed. In ACPiece, these emotions are termed “pieces”. The program enhances communication among care managers who excel at recognizing the emotions of long-term care service users but often find it challenging to address future healthcare decisions (Table 1).

### 2.4. Survey Procedure

A total of 30 participants engaged in ACP conversations with care managers and completed questionnaires on ACP engagement and PTSD in the original study. The study protocol was finalized by August 2022, and study information was obtained from the University Hospital Medical Information Network in Japan (study ID: 000048573) [14]. The research commenced on 15 September 2022. The final participant was recruited on 8 December 2022, with follow-ups concluding on 26 January 2023; the findings were published on 23 September 2024 [13]. In the present study, we conduct a secondary analysis using the raw data from this published paper, focusing specifically on frailty and PTSD.

### 2.5. Participants

Participant details are included in the previous report [13]. To summarize, nine care managers participated in ACP discussions with 30 users receiving long-term care services after completing the ACPiece program. Each of the nine care managers selected three to four users through convenience sampling. The criteria for including long-term care service users were as follows: users assigned to a care manager, those over 65 years of age, individuals capable of communicating about ACP, users with family members available to discuss ACP, and those with a healthcare provider who could engage in ACP discussions. The exclusion criteria included users with a previous history of ACP, those considered mentally unstable by their care managers and unsuitable for ACP intervention or surveys, users whose physical conditions made ACP undesirable, and those whose care managers’ interventions had lasted less than 12 weeks. Care managers were careful not to select users under a lot of stress based on this criterion. Users with prior ACP experience were excluded to prevent adjustments based on their past ACP involvement. Despite efforts to exclude mentally unstable users, participants with high PTSD scores were included, as their inclusion could not be avoided.

Table 2 shows the characteristics of long-term care service users and care managers. Further details can be found in a previous study [13]. In summary, 15 (50%) long-term care service users exhibited a CFS score of 1–4, whereas 15 (50%) exhibited a CFS score of 5–9. All 30 long-term care service users completed an approximately 60 min ACP conversation with their care managers. Although a second ACP conversation was not prohibited, only one ACP conversation was conducted. Care managers consistently took great care to ensure that communication remained stress-free for users during the conversation regarding advocate selection, life-prolonging treatment choices, user values, matters related to their lives, and relationships with their family members.

### 2.6. Questionnaires and Measurement

The questionnaire and measurements are detailed in a previous study [13]. The primary endpoint, the ACP engagement score [35,36], was measured both before and after the ACP discussion, while the secondary endpoint, the Impact of Events Scale-Revised (IES-R) score [37,38], which assesses PTSD, was evaluated following the ACP discussion. The IES-R is a 22-item questionnaire tested for reliability and validity in Japanese; it includes eight items for intrusion, eight for avoidance, and six for hyperarousal. For internal consistency, Cronbach’s alpha must range from 0.92 to 0.95 for the upper scale and from 0.80 to 0.91 for the lower scale [38]. Given that this was a pilot study, the sample size was determined to be 30 participants to ensure statistical validity [39,40].

### 2.7. Statistical Analysis

Data were analyzed for participants who agreed to participate in the study and who completed questionnaires before and after the ACP discussion with the care manager. There were no missing data. Continuous variables are presented as means and standard deviations, while categorical variables are presented as frequencies and percentages.

In the previous study [13], data from the IES-R of 30 participants, measured as secondary endpoints, were analyzed as follows. First, the relationship among the CFS [34], a representative rating scale for frailty, and the IES-R, a representative rating scale for PTSD, was examined. The participants were classified into two groups based on the CFS score, inferring from past literature that CFS scores above or below a certain threshold have different clinical relevance [13,25,26,30]. For effect sizes, an effect size, r, that could be adapted to nonparametric data was measured, with an effect size of ≥0.1 considered small, ≥0.3 medium, and ≥0.5 large [41]. Statistical significance was set at *p* < 0.05. In addition, the median, minimum, maximum, first and third quartiles, and 95% confidence intervals are presented. For the sensitivity analysis, the Mann–Whitney U and Brunner–Munzel tests were conducted. Second, logistic regression analysis was performed to determine whether the IES-R was affected by confounding factors other than CFS. The IES-R scores were dichotomized using the median score as the reference. Lastly, a hierarchical cluster analysis was used for the sensitivity analysis. Participants were divided into statistically significant groups, the clinical significance of each group was examined, and multiple comparisons between the groups were made. For multiple comparisons, the Tukey method was used for parametric data and the Kruskal–Wallis method was used for nonparametric data.

Statistical analyses were performed using Microsoft Excel 2016 MSO (version 2022) provided by Microsoft Corporation, USA and EZR version 1.55 [42]. The reporting for this study conformed to the guidelines of the Standards for Quality Improvement Reporting Excellence (SQUIRE 2.0) [43]. The checklists developed according to these guidelines are presented as the Supplementary File S1.

## 3. Results

**Figure 1:**
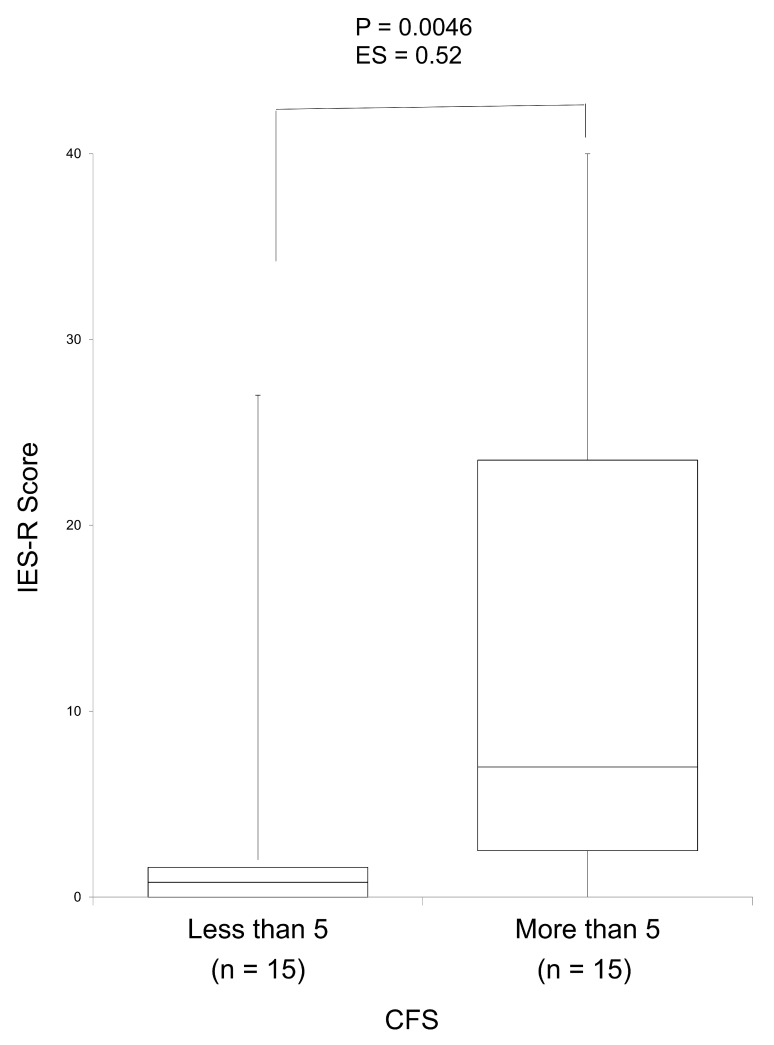
Relationship between the CFS and IES-R as a measure of post-traumatic stress disorder using a box-and-whisker plot. The group with a CFS of greater than 5 had a statistically significantly higher IES-R score than the group with a CFS lower than 5 (*p* = 0.0046). The effect size was also large (ES = 0.52). The maximum, third quartile, median, first quartile, minimum, mean, standard deviation, and 95% confidence intervals for the group with a CFS lower than 5 were 27.0, 2.0, 1.0, 0.0, 0.0, 4.1, 8.5, and −6.3–8.8, respectively, while they were estimated at 40.0, 23.5, 7.0, 2.5, 0.0, 13.4, 13.2, and 6.1–20.7, respectively, for the group with a CFS greater than 5. ES, effect size; SD, standard deviation; 95% CI, 95% confidence interval; IES-R, Impact of Event Scale-Revised; CFS, Clinical Frailty Scale.

Figure 1 shows the relationship between the CFS and the IES-R as a measure of PTSD. Notably, the IES-R score was significantly higher in the group with a CFS of ≥5 than in the group with a CFS of <5. The effect size was large. IES-R scores were not measured before ACP communication between users and care managers but only after.

Table 3 presents the logistic regression analysis results with a dichotomized IES-R score of <3 or ≥3 as the objective variable. A CFS score of ≥5 was associated with a higher IES-R score. The other explanatory variables were assumed to have no apparent effect on the IES-R scores.

Four groups were selected from the hierarchical cluster analysis based on users with increasing mean IES-R scores and the percentage of users with a CFS of ≥5. Multiple comparisons among these groups revealed the following characteristics. Group 1 consisted of younger users with longer relationships with care managers. Group 2 showed no obvious characteristics. Group 3 comprised older care managers. Group 4 consisted of care managers with fewer years of experience (Table 4).

## 4. Discussion

This is the first study to reveal an association between the degree of PTSD in long-term care service users and their degree of frailty, based on an ACP conversation between users and their care managers. The IES-R score, which determines the level of PTSD, was not measured before ACP communication between users and care managers, but only after.

The most important finding of this study is the possibility of predicting high PTSD scores in users, who could not be excluded from ACP conversations in advance or terminated mid-conversation, based on their degree of frailty, which can be assessed relatively quickly. Specifically, care managers find it difficult to exclude such users in advance or terminate ongoing ACP conversations due to the characteristics of Japanese people, who value their relationships with others and may not express their feelings clearly [4]. In Japan, culture is a complex context, and users want care managers to understand their true emotions without having to express them. Thus, even when users perceive that they are under stress and do not want to communicate with their care manager, they may want the care manager to understand their true emotions without reporting them to the care manager [44]. This trend is uncommon in Western countries [44]. Although previous studies have discussed the relationship between frailty and PTSD [15,16], no studies have examined their relationship during ACP conversations with a care manager. In this study, we found a relationship between frailty and PTSD during ACP conversations between users and their care managers. Notably, PTSD scores are high among relatively young users with advanced frailty.

The second important finding is the addition of evidence on the PTSD scores of long-term care service users themselves, rather than those of the surrogate decision-makers. Although previous studies have focused on the PTSD scores of surrogate decision-makers after ACP [17,18,19,20], none have examined the PTSD scores of care service users themselves. Our study found an association between the PTSD scores of care service users after ACP and their degree of frailty. This finding is more important in Japan than in Western countries. This is because, in Japanese culture, understanding the true emotions of long-term care service users is challenging [44].

Interestingly, our study revealed that the CFS can serve as an indicator of both the benefits and disadvantages of ACP. Although previous research has reported that a CFS score of ≥5 promotes ACP engagement [13], the present study suggests that a CFS score of ≥5 may result in a high IES-R score, which may be a disincentive to engage in ACP. Studies have also reported CFS as a good indicator of ACP initiation [24,25], and that higher CFS scores are associated with higher rates of ACP initiation [26]. Additionally, conservative treatment with ACP is more frequently chosen in the CFS group [27]. Our results indicate that a CFS score of ≥5 is a good indicator for ACP initiation and that care managers should be mindful of not stressing care service users during these conversations. Therefore, a high CFS score can be a factor that both promotes and inhibits ACP. This finding aligns with the conclusions of previous studies [28,29], which indicate that the presence or absence of a CFS assessment, as well as a CFS score, does not necessarily lead to ACP implementation. Therefore, we must continue daily stress-free communication training for long-term care service users, using the CFS as a communication guide during ACP discussions.

One of the key strengths of this study is that it fills a research gap in community-based ACP intervention studies led by care managers. Our findings demonstrate that ACP conversations can be effectively initiated using CFS, a relatively easy-to-assess indicator, as a guidepost. Establishing procedures to use CFS assessments in ACP communication to reduce psychological stress is essential. The implications of the main findings for practical use are as follows: users with a CFS of ≥5 can receive more personalized care through more careful communication. However, it is necessary to clarify the type of communication that is careful.

Future research should first include a qualitative study exploring the stress associated with ACP conversations between long-term care service users and care managers based on the degree of users’ frailty. In addition, a randomized controlled trial should be conducted with respect to ACP conversations with care managers trained in the ACPiece program as the intervention group, stratified by CFS values of <5 and ≥5, as both the existing literature [13] and our study indicate that adjustments for confounding factors are needed.

A small sample size could be a limitation of this study. Furthermore, as this intervention study did not include a control group, it may have introduced a selection bias among the long-term care service users who participated. Additionally, we did not measure stress levels before the ACP intervention.

## 5. Conclusions

This study highlights that the degree of PTSD in long-term care service users is associated with their degree of frailty, as revealed through ACP conversations between the users and their care manager. The main finding of this study is that the degree of frailty among long-term care service users may predict stress due to ACP conversations. The implications of the main findings for practical use are as follows: users with a Clinical Frailty Scale score of ≥5 can receive more personalized care through more careful communication. We should conduct a qualitative study on what constitutes more careful ACP communication and then conduct comparative research stratified by CFS values of ≥5 vs. <5.

## Figures and Tables

**Table 1:** Contents of the ACPiece program.

Contents	Time (min)
1. Presentation of an overview of ACP	15
2. Role-playing to practice skills in repetition and silence	40
3. Engaging in training to identify ACP-related terms and expressions from the case scenario’s life story	25
4. Role-playing to initiate ACP	40
5. Role-playing to understand anxiety, concerns, personal values, and life perspectives	40
6. Role-playing to determine who will act as the person’s advocate	40
7. Role-playing to assess how much authority the person wishes to give to the advocate	55
8. Role-playing to discuss future healthcare decisions with the individual and their advocate	70
9. Group activity to explore ethical strategies when there is a clash of values and opinions between the care professional and the individual and their advocate	60

ACP, advance care planning.

**Table 2:** Characteristics of long-term care service users and care managers.

Characteristics of Care Managers and Long-Term Care Service Users	
Characteristics of long-term care service users (*n* = 30)	
Age (years), mean (SD)	81.9 (8.2)
Sex, *n* (%)	
Male	7 (23.3)
Female	23 (76.7)
Number of family members living together, *n* (%)	
One person	14 (46.7)
More than two persons	16 (53.3)
Clinical Frailty Scale score, *n* (%)	
1–4	15 (50.0)
5–9	15 (50.0)
Characteristics of the care managers (*n* = 9)	
Age (years), mean (SD)	53.3 (8.9)
Years of care manager experience, mean (SD)	13.9 (3.9)
Characteristics of care managers (*n* = 9) and long-term care service users (*n* = 30)	
Years of shared commitment, mean (SD)	4.8 (3.3)

The Clinical Frailty Scale is a nine-point score by Rockwood et al. [34]. SD, standard deviation.

**Table 3:** Logistic regression analysis using Impact of Events Scale-Revised as the objective variable (*n* = 30).

Variable	Analysis with All Explanatory Variables				Variable Selection Using the Stepwise Method			
Explanatory Variable	Odds Ratio	95% CI		*p*-Value	Odds Ratio	95% CI		*p*-Value
		Lower Limit	Upper Limit			Lower Limit	Upper Limit	
Intercept	0.00	0.00	820.00	0.20	0.00	0.00	0.51	0.03
Age of users	1.05	0.91	1.21	0.54				
Sex	8.05	0.67	96.90	0.10	6.60	0.63	69.40	0.12
CFS	9.25	1.07	79.60	0.04 *	13.10	1.76	97.40	0.01 *
Living alone	1.03	0.12	8.67	0.98				
Years of experience as a care manager	1.39	0.90	2.14	0.14	1.24	0.92	1.67	0.16
Questionnaire interval	1.05	0.94	1.19	0.39				
Duration of the relationship	0.87	0.63	1.21	0.41				

The Clinical Frailty Scale is a nine-point scale developed by Rockwood et al. [34]. The continuous variables included were users’ age, care managers’ age, years of care manager experience, questionnaire interval, and years of involvement between long-term care service users and care managers. Sex was coded as 0 for males and 1 for females. The CFS was coded as 0 for <5 and 1 for ≥5. The living alone status was coded as 0 if the patient did not live alone and 1 if the patient lived alone. The objective variable, the IES-R score, was dichotomized based on the median value: an IES-R score of <3 was defined as 0 and a score of ≥3 as 1. VIF values for the users’ age, sex, CFS, living alone, years of experience as a care manager, interval, and duration of the relationship were, in order, 1.82, 1.30, 1.28, 1.22, 1.81, 2.34, and 1.47, respectively, indicating no multicollinearity issues. The correlation coefficient between the care manager’s age and years of experience was 0.58, and the VIF values were 3.67 and 4.78, respectively, indicating that multicollinearity was not negligible. Therefore, the age of the care manager was excluded from the logistic regression analysis. An IES-R score of more than 3 was influenced only by a CFS score of more than 5 and not by any other explanatory variables. The results were similar when using a stepwise method. * *p* < 0.05. 95% CI, 95% confidence interval; VIF, variance inflation factor.

**Table 4:** Multiple comparisons among the four clusters identified through hierarchical cluster analysis (*n* = 30).

	Group 1 (*n* = 3)	Group 2 (*n* = 6)	Group 3 (*n* = 13)	Group 4 (*n* = 8)
IES-R score, mean (SD)	35.3 (3.68)	20.5 (5.97)	2.2 (2.04)	0.5 (0.71)
Users with a CFS of ≥5, number (%)	3.0 (100.00)	4.0 (66.70)	6.0 (46.15)	2.0 (25.00)
Age of users, mean (SD)	69.3 (4.99) ^†^	85.0 (4.76)	83.2 (8.80)	82.3 (5.43)
Age of care manager, mean (SD)	49.0 (0.00)	53.2 (7.45)	61.1 (3.25) ^‡^	42.3 (3.15)
Years of care manager experience, mean (SD)	16.0 (0.00)	16.3 (5.68)	14.7 (1.73)	10.0 (2.60) ^§^
Years of the relationship, mean (SD)	9.7 (4.03) ^¶^	2.7 (0.75)	3.7 (2.13)	6.4 (3.35)

Hierarchical cluster analysis was conducted to identify the four groups, and the clinical significance of each group was examined. The results showed that the mean IES-R score of users and the percentage of users with a CFS of ≥5 increased progressively from Group 1 to Group 4. ^†^ Users in Group 1 were younger than those in Groups 2 and 3 (*p* = 0.031 and 0.035, respectively). ^‡^ Care managers in Group 3 were older than those in Groups 1, 2, and 4 (*p* = 0.036, 0.042, and <0.001, respectively). ^§^ Care managers in Group 4 had fewer years of experience than those in Group 3 (*p* = 0.001). ^¶^ The duration (in years) of the user–care-manager relationship was longer in Group 1 than in Groups 2 and 3 (*p* = 0.007 and 0.012, respectively).

## Data Availability

Upon reasonable request, the datasets that were created and/or examined in this study can be obtained from the corresponding author.

## Supplementary Materials

The following supporting information can be downloaded at: https://www.mdpi.com/article/10.3390/jpm15040159/s1, File S1: Reporting checklist for quality improvement in health care.

## Abbreviations

The following abbreviations are used in this manuscript:

ACP	Advance care planning
PTSD	Post-traumatic stress disorder
CFS	Clinical Frailty Scale
IES-R	Impact of Events Scale-Revised
